# Antibacterial Effect of Phenylboronic Acid on *Escherichia coli* and Its Potential Role as a Decontaminant of Fresh Tomato Fruits

**DOI:** 10.17113/ftb.63.01.25.8771

**Published:** 2025-03

**Authors:** Branka Bedenić, Katarina Martinko, Edyta Đermić, Lovorka Vujić, Siniša Ivanković, Mladen Miloš, Isidoro Feliciello, Damir Đermić

**Affiliations:** 1 Biomedical Research Center Šalata-BIMIS University of Zagreb School of Medicine, Department for Clinical Microbiology and Infection prevention and Control, University Hospital Centre Zagreb, Croatia; 2 University of Zagreb Faculty of Agriculture, Division of Phytomedicine, Department of Plant Pathology, Zagreb, Croatia; 3 University of Zagreb Faculty of Pharmacy and Biochemistry, Zagreb, Croatia; 4 Ruđer Bošković Institute, Division of Molecular Medicine, Zagreb, Croatia; 5 Faculty of Chemistry and Technology, University of Split, Split, Croatia; 6 Department of Clinical Medicine and Surgery, University of Naples Federico II, Napoli, Italy; 7 Ruđer Bošković Institute, Division of Molecular Biology, Zagreb, Croatia

**Keywords:** *Shigella sonnei*, *Salmonella enteritidis*, *Yersinia enterocolitica*, multidrug-resistant *Escherichia coli*, ESBL

## Abstract

**Research background:**

Food safety is threatened by the contamination of fresh fruits and vegetables by pathogenic bacteria, among which the particularly widespread ones are coliform bacteria. Due to the continuous increase in the incidence of severe diseases caused by the consumption of fresh (tomato) fruits contaminated with *Escherichia coli*, antimicrobial postharvest measures are needed. The problem is that many active antimicrobial compounds have a weak and short-lasting effect and/or are not environmentally friendly. Recently, the antibacterial and antifungal activity of environmentally friendly agent phenylboronic acid (PBA), including on two tomato pathogens, has been reported.

**Experimental approach:**

The aim of this study is to determine the antibacterial effect of PBA on *E. coli* and three enteropathogenic Enterobacterales, and to check its ability to serve as a bacterial decontaminant of fresh tomato fruits.

**Results and conclusions:**

The minimum inhibitory concentration (MIC) of PBA against *E. coli*, as well as *Shigella sonnei*, *Salmonella enteritidis* and *Yersinia enterocolitica* was 1.0, 1.2, 1.0 and 0.8 mg/mL, respectively. In addition, we have shown that PBA has a bacteriostatic effect on *E. coli* at lower concentrations and a bactericidal effect at higher (>3.0 mg/mL) concentrations. Importantly, the study found that an *E. coli* strain resistant to seven commonly used antibiotics, as well as strains producing extended-spectrum beta-lactamases (ESBL), is as sensitive to PBA as the wild-type strain without any resistance, suggesting that the mechanism of action of PBA differs from that of all these antibiotics. Finally, we have shown that washing and incubating contaminated tomato fruits in PBA solution reduces the growth of *E. coli* washed from fresh tomato fruits in a concentration- (0.5–3.0 mg/mL) and time-dependent manner, while having no adverse effect on the tomato fruits.

**Novelty and scientific contribution:**

This is the first report on the antibacterial effect of PBA on medically important bacteria *E. coli, S. enteritidis, S. sonnei *and* Y. enterocolitica*. Moreover, we show that PBA kills multidrug-resistant *E. coli,* including those producing ESBL, making it a promising agent against such bacteria. Finally, PBA is shown to be an effective decontaminant of *E. coli* on fresh tomato fruits.

## INTRODUCTION

Contamination of fresh fruits and vegetables by pathogenic bacteria constitutes a public health risk, which makes it a permanent challenge for the modern food industry (*1*, *2*). Among the most common contaminants of fresh food is *Escherichia coli* (*3*), the consumption of which results in various gastrointestinal infections, as it is a dangerous human pathogen (*1*, *4*). For instance, a large outbreak of *E. coli* O104:H4 occurred in Germany in 2011, affecting 3842 people and resulting in 53 deaths, which was attributed to the consumption of contaminated fresh bean sprouts (*5*). The bacterium is a Gram-negative, straight and rod-shaped (*6*). Being a facultative anaerobe, *E. coli* can survive in the absence of oxygen (*7*), which increases the risk of food contamination (*1*). *E. coli* is an indicator of faecal contamination and water pollution (*8*, *9*). Thus, irrigation water contaminated with *E. coli* supplied to crops is a potential source of contamination of fresh fruits and vegetables (*9*, *10*) by coming in contact with the plant foliage or wounds (*11*) through bioaerosols generated by sprinkler irrigation (*12*). Solomon *et al.* (*13*) state that such a mode of spread increases the population of *E. coli* on fresh fruits the most. Among the cultivated plants, tomatoes have one of the highest risks of contamination by *E. coli* (*10*).

Fresh tomato fruits are occasionally contaminated with pathogens, which results in foodborne diseases and epidemics. Guo *et al.* (*14*) reported that in 1990, 176 cases in humans, caused by the consumption of raw tomato fruits, were reported in Illinois, Michigan, Minnesota, and Wisconsin, USA. Due to the continuous increase in diseases transmitted by consumption of contaminated raw vegetables such as tomatoes, effective antimicrobial methods are needed during processing of harvested fruits (*15*).

A recent report (*2*) describes the decontamination of the *E. coli* population on the surface of fruits using various compounds with antimicrobial activity. Although effective in reducing cross-contamination of fruits with *E. coli*, some decontaminating agents are of limited utility because their effectiveness decreases rapidly (*16*) and some are explosive or irritant (*17*). An alternative approach to decontamination of *E. coli* from tomatoes involves using environmentally friendly and highly efficient compounds at low concentrations (*2*). We have recently reported the antibacterial and antifungal activity of phenylboronic acid (PBA) against tomato pathogens at the concentrations that are not toxic to the plant (*18*-*20*). This makes PBA a suitable candidate for the decontamination of fresh fruits, especially since PBA is well tolerated by mammals (*21*, *22*) and is considered environmentally friendly (*20*, *23*, *24*). PBA is a derivative of the medically important boric acid (*25*), which in certain concentrations has a significant antimicrobial effect on some medically important bacteria (*26*). However, its activity on *E. coli* and its relatives from the Enterobacteriaceae family, such as *Salmonella enteritidis, Shigella sonnei *and* Yersinia enterocolitica* has not been reported yet. Therefore, in this study, we have determined the PBA MIC for these common causative agents of foodborne illnesses, as well as the *in vitro* effect of PBA on *E. coli* growth and viability, including on multidrug-resistant strains. Finally, we determined the PBA inactivation of *E. coli* washed from the surface of fresh tomato fruits.

## MATERIALS AND METHODS

### Bacterial strains

We used the wild-type K-12 strain MG1655, which is a commonly used laboratory strain close to the archetypal *E. coli* K-12 strain (*27*). It has no antibiotic resistance and is non-pathogenic. We have constructed an MG1655 derivative DE728 resistant to seven commonly used antibiotics: tetracycline, ampicillin, chloramphenicol, kanamycin, rifampicin, streptomycin and nalidixic acid. Antibiotic resistance was produced either by selecting forward mutations of the *E. coli* genes *rpsL*, *gyrB* and *rpoB* for streptomycin, nalidixic acid and rifampicin resistance, respectively, or by the introduction of transposon-marked alleles by P1 phage transduction (*28*): *thr*::Tn*10*, *zoi*::Tn*3*, *malB*::Tn*9* and *ΔproA*::Km, which confer resistance to tetracycline, ampicillin, chloramphenicol and kanamycin, respectively. *Salmonella enteritidis, Shigella sonnei Yersinia enterocolitica* and *E. coli* strains that produce extended-spectrum beta-lactamases (ESBL) come from the collection of the Department of Medical Microbiology and Parasitology of the School of Medicine, University of Zagreb, Zagreb, Croatia.

The *E. coli* strains that produce ESBL were clinical isolates from urine (Table S1) as described previously (*29*).

### Determination of minimum inhibitory concentration of PBA

The minimum inhibitory concentration (MIC) of phenylboronic acid (PBA) for *E. coli*, *S. enteritidis, S. sonnei* and *Y. enterocolitica* was determined by agar dilution according to CLSI standards (*30*). An inoculum of 10^4^ CFU per spot was applied on a Luria-Bertani (LB) agar (Gibco, Waltham, MA, USA) plate containing a certain concentration of PBA, which was then incubated for 24 h at 37 °C. MIC was defined as the lowest concentration inhibiting the growth of colonies on agar. As a control, plates without PBA were used and the normal growth and the titre of viable bacterial cells was determined.

### Preparation of PBA concentration range

Based on the determined MIC for *E. coli*, PBA (Merck, Rahway, NJ, USA) was prepared in a range of concentrations (1/2 MIC, 1 MIC, 2 MIC and 3 MIC). A stock solution of PBA at a concentration of 10 mg/mL was prepared in sterile water or LB agar, which was then diluted to the final concentrations from 0.4 to 4.0 mg/mL.

### Determination of growth kinetics and viability of E. coli treated with PBA

Following a previously described procedure (*18*), *E. coli* was grown in a liquid LB medium at 37 °C with aeration. The bacterial culture in the exponential growth phase was diluted 10-fold into the fresh LB medium containing PBA. The bacteria were incubated in the PBA-enriched medium at 37 °C with aeration, and the samples were periodically taken. Their absorbance (*A*_600 nm_) and viable count were determined by a colorimeter (Novaspec II; Amersham Pharmacia Biotech, Amersham, UK). The titre of viable bacteria (either wild-type or its derivative resistant to: tetracycline, ampicillin, chloramphenicol, kanamycin, rifampicin, streptomycin, and nalidixic acid) was determined by a serial dilution of bacterial cultures in 67 mmol/L phosphate buffer and plating them on LB agar plates without PBA, which were incubated at 37 °C for 48 h. The absorbance and the titre of viable cells at the start of incubation were used as a reference for expressing their changes during incubation.

### PBA treatment of tomato fruits contaminated with E. coli

Considering the determined MIC, *E. coli* was tested against a range of MIC concentrations according to the modified method of inactivation of *E. coli* by washing from fresh tomato fruits as described by Zhang *et al*. (*2*). Thirty-six cherry tomato (*Solanum lycopersicum* var. *cerasiforme*) fruits were immersed in a suspension of *E. coli* (7.9·10^8^ CFU/mL) and soaked for 30 min. After soaking, the fruits were dried on a paper towel to facilitate adhesion of bacteria to the fruit surface. Each tomato fruit was then placed in a sterile plastic box (8 cm×6 cm×7 cm) and the prepared PBA solution was added. The sealed box containing the fruit immersed in a previously determined concentration of PBA was placed on a shaker and secured with adhesive tape. The fruit was washed with the PBA solution by shaking (1000 rpm/2 min). The procedure was repeated with control solutions of sterile distilled water and ethanol (1.0 %). After rinsing, the solution in which the tomato fruits had been washed was pipetted in a volume of 100 µL onto LB medium in sterile Petri dishes (9 cm) and spread evenly with a glass plate spreader. The inoculated Petri dishes were incubated in an air chamber at 37 °C in the dark.

The experiment was altered by prolonging the incubation of tomato fruits in PBA solutions for 120 min. The duration of exposure was determined by a preliminary experiment (data not shown), which determined that there was no cracking or discoloration of the tomato fruits after 120 min of immersion in PBA.

The results were read 72 h after setting up the experiment by photographing the grown colonies in Petri dishes. The total area (cm^2^) of *E. coli* colonies was measured using the software ImageJ according to Guzmán *et al*. (*31*) to determine the degree of pathogen inactivation.

Statistical data were analysed by one-way analysis of variance (one-way ANOVA) and differences between treatments were evaluated using the Tukey’s test (p≤0.05) (*32*) and SPSS v. 27 (*33*).

## RESULTS AND DISCUSSION

### Determination of PBA minimum inhibitory concentration

The growth of bacterial colonies was monitored on the plates with concentrations ranging from 0.7 to 2.5 mg/mL PBA. We analysed the common human pathogenic Gram-negative bacteria that are the most prevalent causative agents of foodborne infections such as *E. coli* and its relatives *S. enteritidis, S. sonnei* and *Y. enterocolitica*. As shown in Table 1, the minimum concentrations of PBA that blocked the growth of *E. coli*, *S. enteritidis, S. sonnei* and *Y. enterocolitica* were 1.0, 1.0, 1.2 and 0.8 mg/mL, respectively. From this we infer that these concentrations are the MICs of PBA for these bacteria. The observed PBA MICs are similar to that of their plant pathogenic relative *Erwinia amylovora* (0.8 mg/mL) and about twice as high as the PBA MIC for another plant pathogenic bacterium *Pseudomonas syringae* pv. *tomato* (0.5 mg/mL) (*18*).

**Table 1:** Inhibitory effect of phenylboronic acid (PBA) on the growth of human pathogenic bacteria on agar plates after 24 h of incubation at 37 °C

Organism	*γ*(PBA)/(mg/mL)								
	0.7	0.8	0.9	1.0	1.1	1.2	1.5	2.0	2.5
*Yersinia enterocolitica*	+	-	-	-	-	-	-	-	-
*Escherichia coli*	+	+	+	-	-	-	-	-	-
*Salmonella enteritidis*	+	+	+	-	-	-	-	-	-
*Shigella sonnei*	+	+	+	+	+	-	-	-	-

### The effect of PBA on growth and viability kinetics of E. coli

**Fig. 1:**
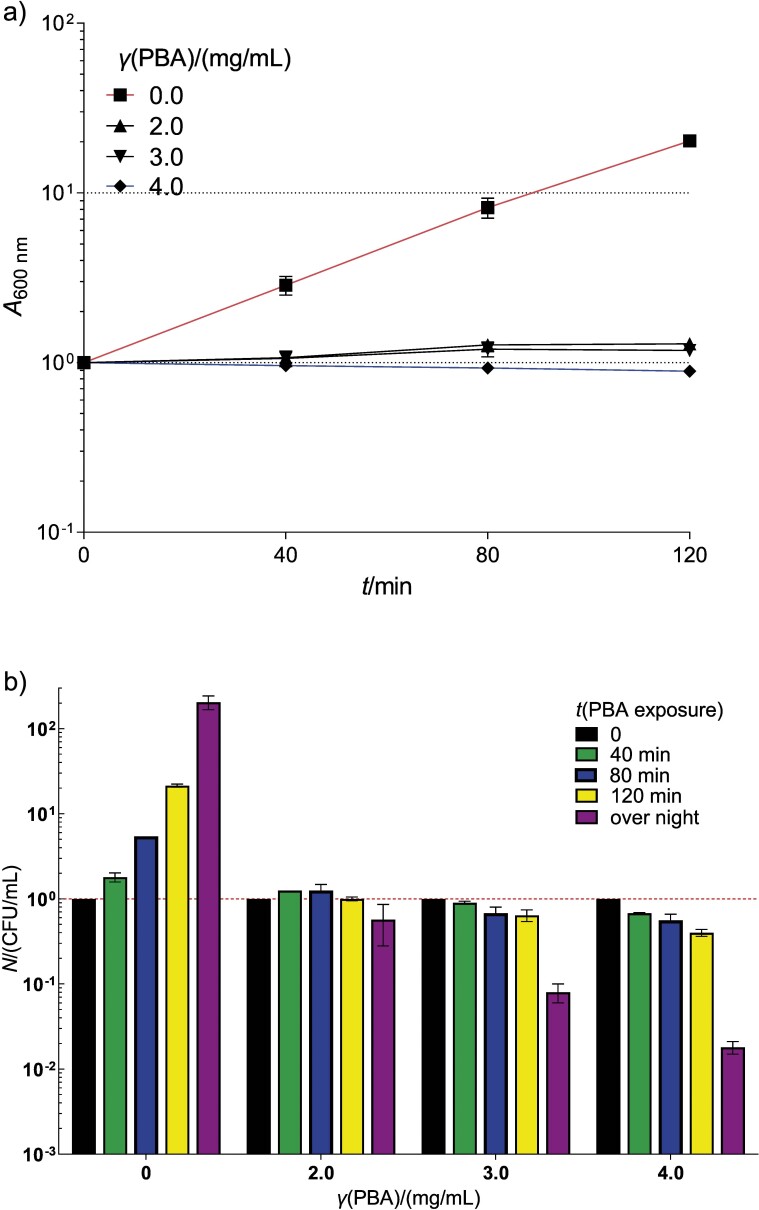
Kinetics of wild-type *E. coli* MG1655 growth (a) and viable cell count (CFU, colony-forming units) (b) in a liquid LB medium supplemented with phenylboronic acid (PBA) at 37 °C. Serial dilution of bacterial cultures was applied on LB plates (with no PBA added) and incubated for 48 h at 37 °C. Absorbance and viable cell titre at the start of incubation were used as a reference for expressing their changes during incubation. Each value is a mean of three independent experiments, with error bars representing standard deviation

Since the MICs of PBA for *E. coli* and its bacterial relatives were similar, we used the former in further research, mainly because of its facultative pathogenic nature and the resulting convenience of working with it, as well as our ample experience in working with this organism. To better characterise the effect of PBA on the physiology of *E. coli*, we measured the growth and survival kinetics of the bacterium in LB medium with different concentrations of PBA. As shown in Fig. 1a, PBA slowed the bacterial growth in a concentration-dependent manner. The mass doubling time of *E. coli* was approx. 26 min in the medium without PBA and increased greatly in the medium containing 2.0 or 3.0 mg/mL PBA, where the bacteria stopped growing. There was a decrease in the absorbance of bacteria in the medium with 4.0 mg/mL PBA (Fig. 1a). These results thus indicate that PBA in concentrations of 2.0 to 3.0 mg/mL has a bacteriostatic effect, while a concentration of 4.0 mg/mL has a bactericidal effect against *E. coli*.

We directly measured the effect of PBA on bacterial viability by determining the viable cell count in cultures containing PBA. In a medium without PBA, the bacterial viable count, expressed as the titre of colony forming units (CFU/mL), grew exponentially (Fig. 1b). On the other hand, in a medium containing 2.0 mg/mL PBA, the titre of viable cells increased only slightly and then fell slightly after prolonged incubation (overnight). The survival of *E. coli* was even more reduced after incubation in the medium with 3.0 mg/mL PBA, which was even more pronounced at a PBA concentration of 4.0 mg/mL, in which case it was reduced about 100-fold after overnight incubation (Fig. 1b). These results show that 2.0 mg/mL of PBA in LB medium has a bacteriostatic effect, while PBA at a concentration of 3.0 mg/mL has a mild bactericidal effect, which strongly increases when the PBA concentration increases to 4.0 mg/mL. The higher PBA concentrations required to inhibit the growth and viability of *E. coli* in this assay are likely due to the much shorter exposure time in the MIC assay. Moreover, the MIC assay does not differentiate between bacteriostatic and bactericidal effects of the tested agent (the effects are added), while the growth and viability kinetics assays are able to differentiate between the two.

### Resistance to multiple antibiotics does not affect E. coli sensitivity to PBA

**Fig. 2:**
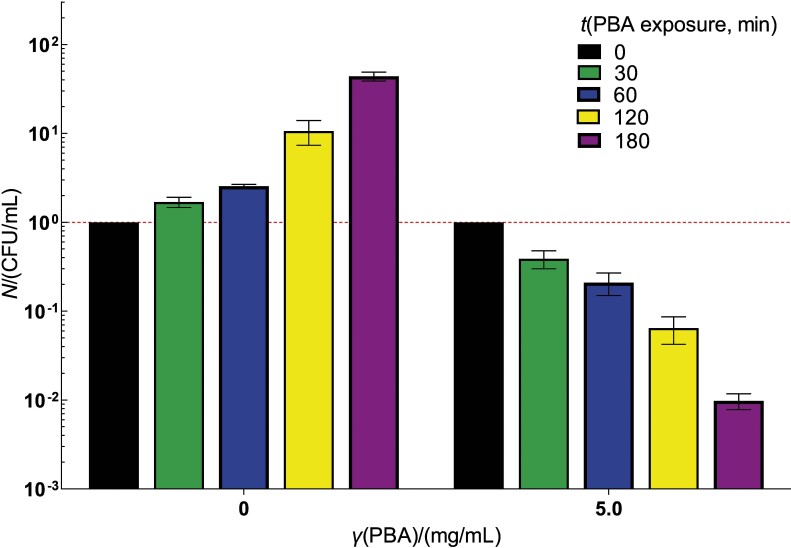
Kinetics of multiple-antibiotic-resistant *E. coli* DE728 viable cell count (CFU) in a liquid LB medium supplemented with phenylboronic acid (PBA), at 37 °C. Bacteria were resistant to tetracycline, ampicillin, chloramphenicol, kanamycin, rifampicin, streptomycin and nalidixic acid. Serial dilution of bacterial cultures was applied on LB plates (with no PBA added) and incubated for 48 h at 37 °C. Viable ell titre at the start of incubation was used as a reference for expressing its changes during incubation. Each value is a mean of three independent experiments, with error bars representing standard deviation

We investigated how resistance to several commonly used antibiotics (*e.g.* tetracycline, ampicillin, chloramphenicol, kanamycin, rifampicin, streptomycin and nalidixic acid) affects the survival of *E. coli* when exposed to PBA. As shown in Fig. 2, the DE728 strain, which is resistant to seven antibiotics, showed a decrease in viable cell titre that is comparable to the survival of its wild-type progenitor (MG1655) when exposed to a similar concentration of PBA (compare Fig. 2 and Fig. 1b). This result indicates that the mechanism of toxicity of PBA to the bacterium differs from that of the seven tested antibiotics, suggesting that PBA can be used against *E. coli* irrespective of its antibiotic resistance and thus represents a valuable alternative for treating infections associated with multidrug-resistant *E. coli* strains. Since the mechanisms of antibiotic activity as well as the mechanisms of bacterial resistance to antibiotics are conserved among different bacteria, the unaffected killing of multidrug-resistant *E. coli* by PBA should be a common feature among bacterial species.

Given the growing problem of increasing antibiotic resistance of medically important pathogenic bacteria, we determined the MIC of PBA against *E. coli* resistant to newer antibiotics, namely strains producing CTX-M beta-lactamases belonging to extended spectrum (ESBL) that are resistant to new-generation penicillin and cephalosporins (Table 2). The problem with ESBL-producing bacteria is that ESBLs are mainly encoded by plasmids, which often also carry genes encoding resistance to other classes of antimicrobials (for example, aminoglycosides, quinolones, tetracyclines, *etc.*) (Table S1) (*29*). This multiple resistance to antimicrobial agents limits the treatment options of ESBL-producing bacteria and poses a risk to successful treatment. As shown in Table 2, the MIC of PBA for all 9 strains producing ESBL was similar to the MIC of the control *E. coli* strain, which has no resistance (1.2 mg/mL). Their MICs varied from 0.8 to 1.3 mg/mL. Our results suggest that PBA can be used against *E. coli* that produce ESBL, thus alleviating the problem of multiple resistance in these bacteria. Further research is certainly needed to optimise the use of PBA in human medicine.

**Table 2:** Inhibitory effect of phenylboronic acid (PBA) on the growth of *Escherichia coli,* producing extended-spectrum beta-lactamases (ESBL), on agar plates after 24 h of incubation at 37 °C

		*γ*(PBA)/(mg/mL)								
Strain	Additional resistance	0.7	0.8	0.9	1.0	1.1	1.2	1.3	1.4	1.5
5	CAZ, CTX, CRO, FEP, GM, CIP	+	-	-	-	-	-	-	-	-
6	CAZ, CTX, CRO, FEP, GM, CIP	+	-	-	-	-	-	-	-	-
11	CAZ, CTX, CRO, FEP, GM, CIP	+	-	-	-	-	-	-	-	-
2	CAZ, CTX, CRO, FEP, GM, CIP	+	+	+	+	-	-	-	-	-
3	CTX, CRO, FEP, GM, CIP	+	+	+	+	-	-	-	-	-
4	CAZ, CTX, CRO, FEP, GM, CIP	+	+	+	+	-	-	-	-	-
1	CAZ, CTX, CRO, FEP, CIP	+	+	+	+	+	-	-	-	-
12	CAZ, CTX, CRO, FEP, CIP	+	+	+	+	+	-	-	-	-
ATCC25922		+	+	+	+	+	-	-	-	-
8	CAZ, CTX, CRO, FEP, CIP	+	+	+	+	+	+	-	-	-

CAZ=ceftazidime, CTX=cefotaxime, CRO=ceftriaxone, FEP=cefepime, IMI=imipenem, MEM=meropenem, GM=gentamicin, CIP=ciprofloxacin, TZP=piperacillin-tazobactam

The decrease in cell density (*A*_600 nm_) in cultures treated with higher concentrations of PBA (>4.0 mg/mL) (Fig. 2) indicates that the mechanism of killing *E. coli* by PBA involves disintegration of the cells. The same effect was observed when *E. amylovora* and *P. syringae* pv. *tomato* were exposed to PBA, albeit at lower concentrations (2.0 and 3.0 mg/mL, respectively) than *E. coli* (*18*). However, the common trait of the effect of PBA on all three bacteria is that the decrease in cell density is observed at about 4 MIC.

### Antibacterial effect of PBA on E. coli washed from tomato fruits

After washing the tomato fruits with PBA at ½ MIC (0.5 mg/mL), 1 MIC (1.0 mg/mL), 2 MIC (2.0 mg/mL) and 3 MIC (3.0 mg/mL), the growth of *E. coli* colonies was measured. The growth area of *E. coli* after washing them with ½ MIC, 1 MIC, 2 MIC and 3 MIC PBA from tomato fruits was reduced by 41, 59, 53 and 85 %, respectively, compared to the control wash with dH_2_O (Table 3).

**Table 3:** Effect of phenylboronic acid (PBA) on the growth of *Escherichia coli* washed from tomato fruits and incubated for either 0 or 120 min in the PBA-containing solutions, compared to control washings with dH_2_O and EtOH (1.0 %) after 72 h incubation

	*A*(colony)/cm^2^					
*t*(incubation)/min			*γ*(PBA)/(mg/mL)			
	dH_2_O	EtOH	0.5	1.0	2.0	3.0
0	(3.4±0.2)^e^	(2.5±0.1)^d^	(2.0±0.2)^c^	(1.4±0.2)^b^	(1.6±0.4)^b^	(0.5±0.2)^a^
120	(3.4±0.2)^d^	(2.5±0.1)^c^	(0.1±0.0)^a^	(1.1±0.1)^b^	(0.2±0.0)^a^	(0.1±0.0)^a^

*Different letters in superscript indicate a significant difference according to the Tukey’s test at the level of p<0.05

Washing *E. coli* cells with ½ MIC, 1 MIC, 2 MIC and 3 MIC of PBA from tomato fruits resulted in a reduction in the growth area of bacterial colonies by 20, 44, 36 and 80 %, respectively, compared to the control wash with EtOH (1.0 %) (Table 3).

The mean values of *E. coli* colony growth for all tested concentrations (½ MIC, 1 MIC, 2 MIC and 3 MIC of PBA) were significantly different from the control washes with dH_2_O and EtOH (1.0 %) according to the Tukey’s test (Table 3). Our results thus show that PBA inhibits the growth of *E. coli* washed from tomato fruits.

### Antibacterial effect of prolonged incubation with PBA on the growth of E. coli washed from tomato fruits

The growth of *E. coli* colonies washed from tomato fruits was recorded after incubation for 120 min with PBA at concentrations of ½ MIC (0.5 mg/mL), 1 MIC (1.0 mg/mL), 2 MIC (2.0 mg/mL) and 3 MIC (3.0 mg/mL).

As shown in Table 3, the growth area of *E. coli* colonies was inhibited by 97, 68, 94 and 97 % after washing with ½ MIC, 1 MIC, 2 MIC and 3 MIC of PBA, respectively, from tomato fruits and after immersion of fruits in the specified concentration range for 120 min, compared to washing with dH_2_O. Washing *E. coli* and exposing the fruits to the indicated concentrations resulted in a 96, 56, 92 and 96 % reduction in bacterial colony growth area compared to the control washing with EtOH (1.0 %).

The mean colony growth values of *E. coli* were significantly different in all tested concentrations (½ MIC, 1 MIC, 2 MIC and 3 MIC PBA) compared to the mean colony growth values in the control samples with dH_2_O and EtOH (1.0 %) according to the Tukey’s test (Table 3).

We can therefore conclude that PBA reduces the growth of *E. coli* washed from tomato fruits in a concentration- and time-dependent manner. Previously, Shen *et al*. (*34*) reported a stronger inhibition of *E. coli* in an aqueous chlorine solution with increasing incubation time. They showed that the inactivation of *E. coli* depends on the efficacy and concentration of the compound and the time of exposure of the bacterium, which is consistent with our results.

Furthermore, the results show a higher efficacy of PBA compared to ethanol, a commonly used disinfectant. Ethanol solution of 1.0 % had a weaker effect on *E. coli* than PBA at 0.5 mg/mL (*i.e.* 0.05 %). It is certainly possible to increase the ethanol volume fraction, but this would potentially have negative side effects since ethanol is a strong oxidizing agent and can therefore have a negative effect on the quality of tomato fruits. On the other hand, PBA is a weak acid, which would therefore weakly affect the tomato fruit even at 1.0 % (we did not observe any adverse effects of PBA at concentrations we used on tomato fruits, data not shown), while we found quite a strong effect on the *E. coli* washed from the tomato fruit already at 0.3 % (3.0 mg/mL) PBA, which means that it is possible to use higher PBA concentrations than those we used here. This is particularly important as PBA is environmentally friendly (*20*, *23*, *24*) and is well tolerated by mammals (*21*, *22*). For instance, the LD_50_ (oral) for a rat for PBA is 0.74 g/kg, compared to the LD_50_ of NaCl, which is 3.0 g/kg (*35*, *36*).

When comparing the results of our *in vitro* experiments, a difference can be observed between the inhibitory effect of PBA dissolved in distilled water during prolonged exposure, where an antibacterial effect on *E. coli* was obtained at 0.5 mg/mL PBA (Table 3), and the inhibitory effect of PBA dissolved in nutrient medium, where an antibacterial effect was obtained at 1.0 mg/mL PBA (Table 1). The discrepancy can be explained by the data of Virto *et al*. (*37*), who showed that the inactivation of *E. coli* by chlorine dissolved in distilled water is significantly more pronounced than the inactivation of bacteria exposed to chlorine in an organic medium. Their results indicate that the bacterial cell membrane is more severely damaged in water than in the organic medium which prevented the permeability of cell membrane and the penetration of chlorine into the *E. coli* cell. Therefore, our results are consistent with that study (*37*).

## CONCLUSIONS

In this study we showed phenylboronic acid (PBA) as a promising antibacterial agent of medical importance due to two of its properties. Firstly, PBA has antibacterial effect against *E. coli* and its enterobacterial relatives. We determined the PBA concentrations with bacteriostatic/bactericidal effects against *E. coli*. Secondly, we have shown that PBA is effective against multidrug-resistant *E. coli*, including resistance to modern antibiotics. Moreover, we used PBA for an efficient decontamination of *E. coli* from fresh tomato fruits, thus disclosing the potential of PBA usage in the decontamination of raw food.

This is the first study of the antibacterial effect of PBA on *E. coli* and its pathogenic relatives, which is supplemented with the determination of practical use of PBA for decontamination of (even multiple-antibiotic-resistant) bacteria on fresh tomato fruits and thus opens up a perspective of PBA application in food processing.

## Appendix

**Table S1:** Additional antibiotic and genomic traits of the clinical isolates of ESBL-producing *Escherichia coli* strains

		MIC/(mg/mL)										DNA profile	
Sample	Strain	CAZ	CTX	CRO	FEP	IMI	MEM	GM	CIP	TZP	Beta-lactamase content	Plasmid type	PFGE cluster
1	3459	32 (R)	16 (R)	128 (R)	2 (I)	0.06 (S)	0.06 (S)	4 (S)	128 (R)	4 (S)	CTX-M-15, TEM-1	X, FIA	E XXIV
2	7377	8 (I)	8 (R)	>128 (R)	64 (R)	0.06 (S)	0.06 (S)	64 (R)	>128 (R)	4 (S)	CTX-M-15, TEM-1	FIA	E XVIIIb
3	16416	4 (S)	16 (R)	128 (R)	4 (R)	0.06 (S)	0.06 (S)	64 (R)	128 (R)	2 (S)	CTX-M-15	X, FIA	E XXXII
4	1358	64 (R)	>128 (R)	>128 (R)	64 (R)	0.06 (S)	0.06 (S)	128 (R)	>128 (R)	32 (S)	CTX-M-15, TEM-1	X	E IX
5	23407	64 (R)	>128 (R)	>128 (R)	64 (R)	0.06 (S)	0.06 (S)	128 (R)	128 (R)	2 (S)	CTX-M-15, TEM-1	L/M	E XXVII
6	3263	16 (R)	128 (R)	64 (R)	64 (R)	0.06 (S)	0.06 (S)	64 (R)	>128 (R)	1 (S)	CTX-M-15	FIA	E II
8	21646	16 (R)	64 (R)	16 (R)	4 (R)	0.06 (S)	0.06 (S)	1 (S)	>128 (R)	0.5 (S)	CTX-M-15	X	E XXVII
11	8537	8 (I)	128 (R)	>128 (R)	64 (R)	0.06 (S)	0.06 (S)	128 (R)	>128 (R)	4 (S)	CTX-M-15	NEG	E XIX
12	4145	128 (R)	>128 (R)	>128 (R)	16 (R)	0.25 (S)	0.25 (S)	2 (S)	32 (R)	2 (S)	CTX-M-15, TEM-1	N	E XIV

ESBL=extended-spectrum beta-lactamase, MIC=minimum inhibitory concentration, CAZ=ceftazidime, CTX=cefotaxime, CRO=ceftriaxone, FEP=cefepime, IMI=imipenem, MEM=meropenem, GM=gentamicin, CIP=ciprofloxacin, TZP=piperacillin-tazobactam. R=resistant, I=intermediately sensitive, S=susceptible. The isolation and characterisation of the *E. coli* strains was reported earlier (*29*)
